# Vacuole Proteins with Optimized Microtubule Assembly Is Required for Fum1 Protein Localization and Fumonisin Biosynthesis in Mycotoxigenic Fungus *Fusarium verticillioides*

**DOI:** 10.3390/jof9020268

**Published:** 2023-02-16

**Authors:** Huijuan Yan, Zehua Zhou, Huan Zhang, Won Bo Shim

**Affiliations:** 1 Department of Plant Pathology and Microbiology, Texas A&M University, College Station, TX 77843, USA; 2 College of Plant Protection & Hunan Provincial Key Laboratory for Biology and Control of Plant Diseases and Plant Pests, Hunan Agricultural University, Changsha 410128, China

**Keywords:** *Fusarium verticillioides*, fumonisins, vacuole, microtubule, tubulins

## Abstract

Fumonisin contamination of corn caused by *Fusarium verticillioides* is a major concern worldwide. While key genes involved in fumonisin biosynthesis are known, the location within the fungal cell where this process occurs has yet to be fully characterized. In this study, three key enzymes, i.e., Fum1, Fum8, and Fum6, associated with early steps of fumonisin biosynthesis pathway, were tagged with GFP, and we examined their cellular localization. Results showed that these three proteins co-localized with the vacuole. To further understand the role of the vacuole in fumonisin B_1_ (FB_1_) biosynthesis, we disrupted two predicted vacuole associated proteins, FvRab7 and FvVam7, resulting in a significant reduction of FB_1_ biosynthesis and a lack of Fum1-GFP fluorescence signal. Furthermore, we used the microtubule-targeting drug carbendazim to show that proper microtubule assembly is critical for proper Fum1 protein localization and FB_1_ biosynthesis. Additionally, we found that α1 tubulin is a negative regulator in FB_1_ biosynthesis. We concluded that vacuole proteins with optimized microtubule assembly play a crucial role in proper Fum1 protein localization and fumonisin production in *F. verticillioides.*

## 1. Introduction

Secondary metabolites are low molecular weight compounds produced by microbes, algae, plants, and animals, which are not essential for maintaining normal growth but are likely important for interaction with other organisms. Examples of such compounds include antibiotics and mycotoxins, which are produced by fungi and can be either beneficial or harmful to humans. Genes responsible for secondary metabolisms biosynthesis and regulation typically occur in clusters [1]. To fully utilize or control these compounds, it is critical to understand where the specific secondary metabolites are synthesized. The possible cellular localization of these biosynthesis enzymes is cytosol, peroxisome, endoplasmic reticulum (ER), golgi, vesicle, and vacuole [2]. To date, subcellular localization of several key secondary metabolites biosynthetic proteins has been studied in filamentous fungi. In *Penicillium chrysogenum*, penicillin biosynthetic pathway enzymes were shown to be in multiple cell compartments including vesicles, vacuoles, peroxisome, and cytosol [3,4,5,6]. In *Aspergillus parasiticus*, aflatoxin biosynthetic enzymes localized to peroxisomes, vesicles, and vacuoles, whereas vesicles play a more critical role in aflatoxin production [7,8,9,10]. In *Fusarium graminearum*, the deoxynivalenol (DON) biosynthetic enzymes are localized in ‘toxisome’, an ovoid and spherical structure composed of the remodeled ER and myosin I-actin cytoskeleton [11,12].

*Fusarium verticillioides* (teleomorph: *Gibberella moniliformis* Wineland) is a fungal pathogen of maize that causes ear rot and stalk rot diseases and thus poses significant risks in food production, quality, and safety. Significantly, *F. verticillioides* produces a range of mycotoxins and biologically active secondary metabolites including fumonisins, fusaric acid, and fusarins in infested maize. While several *Fusarium* species can produce fumonisins, *F. verticillioides* is the most prevalent species with a significant economic impact. A total of 28 fumonisin analogs have been discovered, which are grouped into the A, B, C, and P series of fumonisins [13]. Deeper investigations have been conducted on the B series fumonisin, especially fumonisin B_1_ (FB_1_), since these are known as the most pervasive and toxic forms. Fumonisins are a group of polyketide-derived mycotoxins that share structural similarities with sphinganine and are hazardous to humans and animals [14,15]. Governmental agencies around the world limit the amount of fumonisins in grains and foods intended for human and animal consumption due to health concerns. 

Fumonisins are secondary metabolites with 19–20 carbon backbone polyketide. The fumonisin biosynthesis gene cluster (also known as the “*FUM* cluster”) was initially proposed by Proctor et al. (1999) [16], which is comprised of 16 genes encoding biosynthetic enzymes and regulatory proteins (Proctor et al., 2013). All *FUM* genes, except for *FUM15*-*FUM18*, are critical for fumonisin production in *F. verticillioides*. Specifically, a polyketide synthase (PKS) gene *FUM1*, along with the aminotransferase gene *FUM8*, contribute to the initial stage of fumonisin production. The third step in FB_1_ biosynthesis pathway is thought to be carried out by a cytochrome P450 monooxygenase *FUM6*, whereas Knockout mutants *fum1*, *fum8*, and *fum6* exhibited complete abolishment in FB_1_ production [16,17].

The identification of *FUM* gene cluster allowed researchers to gain a better understanding of the biosynthetic mechanism associated with fumonisins contamination in maize Fusarium ear rot. Previous studies demonstrated that multiple components, environmental and epigenetic factors, that participate in this complex biological process [18,19]. However, the fundamental question of where in *F. verticillioides* cell fumonisins are synthesized remained elusive. Here, our study characterizes the cellular localization of key Fum enzymes in *F. verticillioides*, and how it contributes to FB_1_ production. Specifically, we show that Fum proteins important for early FB_1_ biosynthesis steps co-localized in vacuole with FM4-64 staining. To confirm that Fum proteins co-localizing with FM4-64 are functionally related to the vacuole, we disrupted two genes involved in vacuole functions and monitored Fum1-GFP localization and FB_1_ production. Additionally, with the treatment of carbendazim, a drug that binds tubulin and inhibits microtubule (MT) assembly, we found that microtubule assembly is required for regulating Fum1 protein localization in vacuoles and proper FB_1_ production. Furthermore, we identified α1 tubulin as a negative regulator in FB_1_ biosynthesis.

## 2. Materials and Methods

### 2.1. Strains and Culture Conditions

*F. verticillioides* wild-type (WT) strain M3125 and all mutants were cultured at room temperature on 0.2 × PDA medium. Spores were harvested and determined by a hemocytometer. Minimal medium (MM liquid: 10 g of D-glucose, 50 mL of 20 × nitrate salts, 1 mL Trace elements, 1 mL 1% thiamine, adjust pH = 5.9) and FB_1_ biosynthesis induction medium (myro liquid: 1 g of NH_4_H_2_PO_4_, 3 g of KH_2_PO_4_, 2 g of MgSO_4_·7H_2_O, 5 g of NaCl, 40 g of sucrose per liter, adjust pH = 5.9) were prepared as previously described [20]. A growth assay was performed on V8 agar medium [20]. Each experiment was repeated three times. 

### 2.2. Fum Proteins GFP and mCherry Tagging

A double fusion PCR-based method was used to generate split fragments which targets the native locus in WT strain for Fum1, Fum6, and Fum8 fluorescent strains construction (Figure S1A). Briefly, around 1 kb upstream of stop codon and 3′ flank of the gene of interest were amplified. GFP and hygromycin B phosphotransferase gene (hygromycin B phosphotransferase gene is referred to as *HPH*, *HPH* divided into two parts, named *HP* and *PH* for later *PCR*) were amplified from pKNTG and pBP15 plasmids, respectively. Three fragments (1 kb upstream, GFP and *HP*) were fused by PCR. *PH* and about 1 kb 3′ flanking region were joined as well. Two independent joint-PCR fragments were transformed into WT protoplast. In this way, GFP and *HPH* were inserted to replace the targeted gene stop codon. Targeted genes were expressed under their native promoter and a single copy of the gene existing in the genome. Strains created were Fum1-GFP (FVEG_00316, 2586 aa), Fum6-GFP (FVEG_00317, 1048 aa), and Fum8-GFP (FVEG_14634, 553 aa). The same approach was employed to create Fum6-mCherry in Fum1-GFP background. mCherry and geneticin (*GE* and *EN*) genes were amplified from pKNT-mCherry and pBSG plasmids, respectively. All fragments were amplified by Q5^®^ High-Fidelity DNA Polymerase (New England Biolabs, lpswich, MA, USA). GeneJET Gel Extraction Kit and GeneJET Plasmid Miniprep Kit were used to purify DNA fragments or isolate plasmids in this study (Thermo Scientific, Waltham, MA, USA). Phire plant direct PCR kit (Thermo Scientific, Waltham, MA, USA) was used to confirm the correct single insertion in this study (Figure S1B). All primers used in this study are shown in Table S1.

### 2.3. Construction of FvVam7-mCherry and mCherry-FvRab7 Plasmids

To construct FvVam7-mCherry, *FvVAM7* (FVEG_05495) was amplified with primers Vam7_mcherry-F/R and cloned into PKNT-mCherry, using KpnI and HindIII sites by Gibson assembly (New England Biolabs, lpswich, MA, USA). FvRab7 (FVEG_04809) was tagged with mCherry at the N-terminus. The mCherry was amplified from PKNT-mCherry. All fluorescence constructs in the study used its native promoter and gene fragments amplified from *F. verticillioides* genomic DNA by Q5^®^ High-Fidelity DNA Polymerase (New England Biolabs, lpswich, MA, USA). The purified products were introduced to the HindIII and BamHI sites of pKNT using Gibson assembly (New England Biolabs, lpswich, MA, USA). The plasmid was sequenced and transformed into the Fum1-GFP strain for localization study.

### 2.4. Gene Deletion, FB_1_ and Split Luciferase Complementation Assay

The deletion mutants of ΔFvvam7, ΔFvrab7, ΔFvα_1_ (FVEG_00557), ΔFvα_2_ (FVEG_00855), ΔFvβ_1_ (FVEG_04081), and ΔFvβ_2_ (FVEG_05512) were generated in Fum1-GFP background via split marker approach described before by replacing the gene with the geneticin (Figure S2A) [21]. Respective drug-resistant colonies were screened through two sets of PCR screenings, as depicted in Figure S2B, using the Phire plant direct PCR kit (Thermo Scientific, Waltham, MA, USA). RNA from knockout mutant candidates was extracted and further confirmed by qPCR (Figure S2C). All primers used in this study are listed in Table S1. For FB_1_ assay, autoclaved cracked corn kernels (2 g) were inoculated with spore solutions (200 μL, 10^6^/mL) and incubated at room temperature for eight days [21]. FB_1_ and ergosterol were extracted and determined as previously described [22]. Split luciferase complementation assay was performed as described previously [21,23]. Statistical test was performed using prism software (San Diego, CA, USA, GraphPad Software), and details were shown in the figure legends.

### 2.5. Live Cell Imaging and Staining

Fum1-GFP, Fum6-GFP, and Fum8-GFP spores were grown in 5 mL of MM or myro liquid medium on a 25 mL flask at 28 °C with constant shaking at 150 rpm. The conidia inoculum concentration was 10^5^/mL for this study. Strains were followed by microscopy observation and imaging. For time points study, cultures were assayed at 12, 24, 36, and 48 h. For the FM4-64 staining vacuole assay in Fum1-GFP, mycelia from 48 h culture were stained with 8 μm FM4-64 for 30 min. The cells were washed and observed under a fluorescent microscope. For the FM4-64 staining vacuole assay in WT, ΔFvvam7, ΔFvrab7, ΔFvα1, ΔFvα2, ΔFvβ1, and ΔFvβ2, three-day-old colony was excised from a V8 plate and put on a glass slide with 35 μL of 25 μM FM4-64 for 90 min before observation. Images in this study were prepared using ImageJ software [24].

## 3. Results

### 3.1. Subcellular Localization of Three FB_1_ Biosynthesis Enzymes

**Figure 1:**
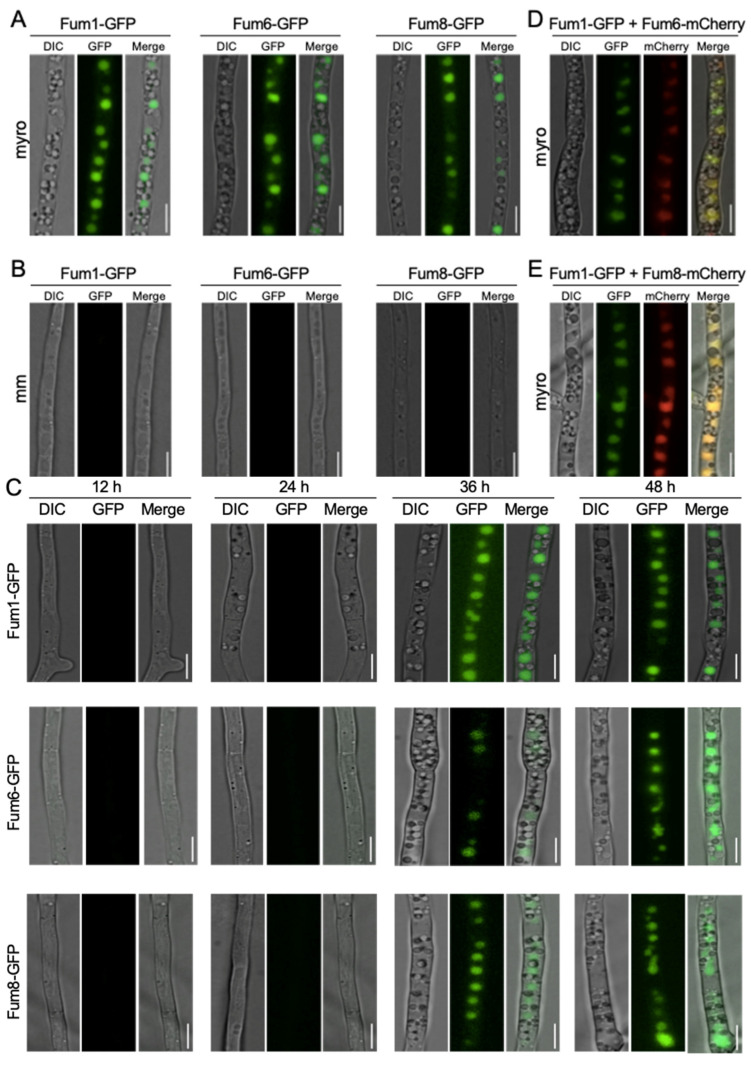
Localization of Fum1, Fum6, and Fum8 in hyphae in toxin inducing and non-inducing media. (**A**) Fum1-GFP, Fum6-GFP, and Fum8-GFP are localized to the spherical structures in hyphae grown in the toxin inducing medium (myro). (**B**) GFP signals were not detected in the minimal medium (mm). All images were taken after 48 h incubation at 28 °C with constant shaking. (**C**) Localization of three key Fum proteins grown in myro broth at different time points (12, 24, 36, 48 h). (**D**) Fum6-mCherry co-localized with Fum1-GFP in hyphae grown in myro liquid medium (**E**) Fum8-mCherry co-localized with Fum1-GFP in myro liquid medium. Bar = 5 μm.

To determine the localization of FB_1_ biosynthesis enzymes, the first three Fum proteins (Fum1, Fum8, and Fum6) in the proposed FB_1_ biosynthetic pathway were studied [18,25]. Fum1, Fum8, and Fum6 were tagged with GFP at their C-terminus under its endogenous locus in *F. verticillioides* (Figure S1). We first employed live-cell imaging to visualize Fum1-GFP, Fum8-GFP, and Fum6-GFP fluorescence under toxin-inducing medium (myro) and toxin non-inducing minimal liquid medium (MM). The results showed that all three tagged Fum proteins were highly induced and localized to punctate structures in hyphae in myro broth, while no green fluorescent was observed in MM after 48 h (Figure 1A,B). To verify that the Fum-protein fluorescence are medium-dependent, we included a SNARE protein tagged strain, Syn1-GFP, from our previous study as a control [26]. Syn1-GFP showed fluorescence signals in both myro and MM (Figure S3). We hypothesized that Fum proteins display different localization at earlier time points. However, our time course study (12, 24, 36, 48 h) demonstrated that Fum1-GFP, Fum6-GFP, and Fum8-GFP localized to similar structures at 36 h and 48 h, but no fluorescence appeared at 12 h and 24 h when cultured in myro medium (Figure 1C). We reasoned that Fum-protein fluorescence first appeared between 24 and 36 h. Indeed, detectable Fum1-GFP, Fum6-GFP, and Fum8-GFP protein fluorescence appeared approximately after 26 h incubation, and fluorescence intensity increased afterwards (data not shown). Additionally, we noted that Fum-GFP signal did not appear in the newly synthesized branching hyphae and apical areas (Figure S4). These data indicate that key Fum proteins Fum1, Fum6, and Fum8 localized to similar punctate structures, and the localizations are regulated in time- and medium-dependent manners.

### 3.2. Co-Localization of Fum1, Fum6, and Fum8 in Vacuoles

**Figure 2:**
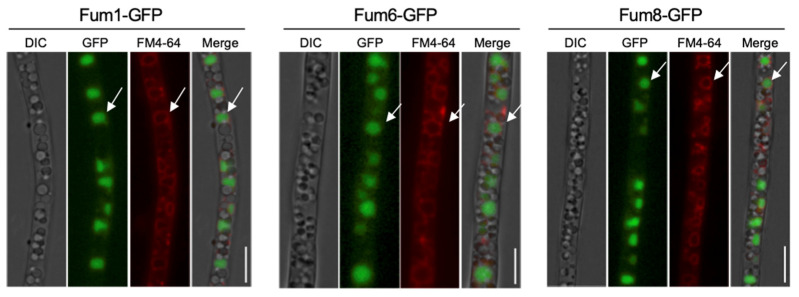
Subcellular localization of Fum1-GFP, Fum6-GFP, or Fum8-GFP with FM4-64 on vacuoles. Fluorescent images indicate co-localization of Fum1-GFP, Fum6-GFP, or Fum8-GFP with FM4-64 labeling on vacuoles. Arrows indicate co-localization. Bars = 5 μm.

Our observation of Fum1, Fum6, or Fum8 localized to similar spherical punctate structures raised the question of whether or not they are expressed in the same organelle. To test this idea, we first tagged Fum6 or Fum8 with mCherry and transformed into Fum1-GFP strain to study the co-localizations of Fum6 or Fum8 with Fum1. As expected, Fum6-mCherry or Fum8-mCherry were co-localized with Fum1-GFP in the same circular organelle (Figure 1D,E). Next, to address the precise structure where Fum proteins localize, we employed lipophilic dye FM4-64 which is well known for staining plasma membranes, Spitzenkörper and vacuolar membrane, with varied incubation time [27]. Fum1-GFP, Fum6-GFP, and Fum8-GFP cells were stained with fluorescent endocytic marker FM4-64 for 30 min for vacuolar membrane stain. We found that Fum1-GFP, Fum6-GFP, and Fum8-GFP overlapped well with FM4-64 labeled vacuoles (Figure 2). In conclusion, our fluorescence microscopic results demonstrated Fum1-GFP, Fum6-GFP, and Fum8-GFP are co-localized with FM4-64 stained vacuoles. 

### 3.3. Two Vacuole Proteins Are Required for FB_1_ Biosynthesis 

**Figure 3:**
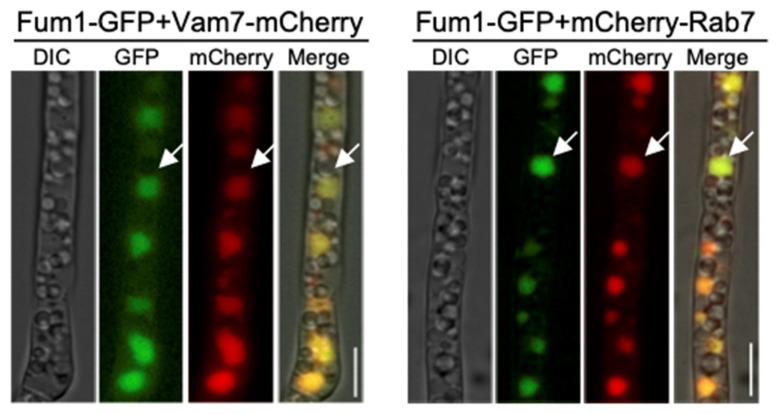
Co-localization of Fum1 with FvVam7 or FvRab7 proteins. FvFum1-GFP was co-localized with FvVam7-mCherry or FvRab7-mCherry in hyphae cultured in myro broth. Images were taken after 48 h constant shaking at 28 °C. Bar = 5 μm.

**Figure 4:**
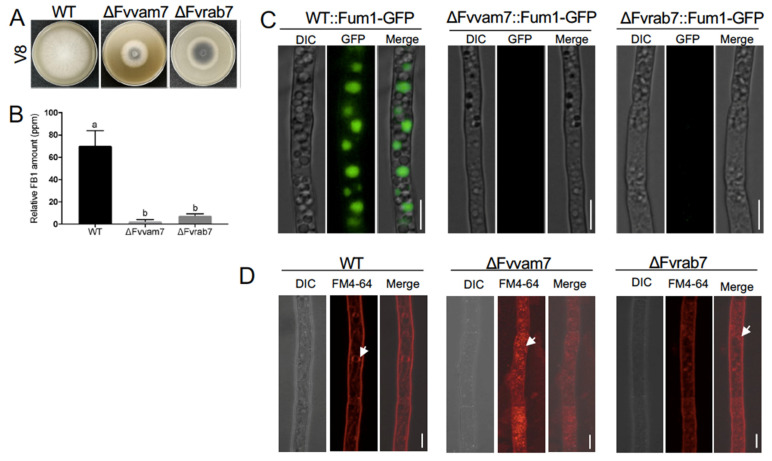
Colony growth and FB_1_ production of ΔFvvam7 and ΔFvrab7 mutants. (**A**) Wild-type (WT), ΔFvvam7, and ΔFvrab7 strains were cultured on V8 agar plates for 7 days at 28 °C. (**B**) FB_1_ levels in WT and mutant strains were normalized with fungal ergosterol levels. WT, Δfvvam7, and ΔFvrab7 strains were grown on autoclaved corn kernels for 8 days at room temperature before assay. Three replicates were performed for each strain. Different letters on the bars indicated significance according to prism Ordinary one-way ANOVA multiple comparisons at *p* < 0.05. (**C**) Fum1-GFP localization was determined in ∆Fvvam7 and ∆Fvrab7 compared to in WT strain. (**D**) FM4-64 was used to stain WT, ∆Fvvam7, and ∆Fvrab7 for vacuoles. Bar = 5 μm.

In *F. graminearum*, two vacuole proteins, FgRab7 and FgVam7, are reported to be localized in the vacuole and essential for vacuole morphology and proper functions [28,29]. We identified these two vacuole protein homologues, FvRab7 and FvVam7, in *F. verticillioides*. To validate our previous localization result from FM4-64 staining, we first expressed FvRab7 and FvVam7 with mCherry in Fum1-GFP background strain. Due to the same location of Fum1, Fum6, and Fum8, we decided to use Fum1 for validation. The results showed that Fum1-GFP displayed co-localization with FvVam7-mCherry and mCherry-FvRab7 on large vacuole structure (Figure 3), which further confirmed the Fum1 localization in the vacuole. Next, we disrupted *FvVAM7* and *FvRAB7* genes in Fum1-GFP strain independently to elucidate the role of the vacuole in the expression of Fum1. Fluorescence microscopy study showed no detectable Fum1-GFP fluorescence signal in ∆Fvvam7 and ∆Fvrab7 mutants in myro medium (Figure 4C), which demonstrated that *FvVAM7* and *FvRAB7* genes are important for Fum1 expression. Furthermore, growth assay showed that ∆*FvVAM7* and ∆*FvRAB7* exhibited severe hyphal growth defect compared to the wild-type progenitor (Figure 4A). Additionally, these two mutants exhibited a small and fragmented vacuole morphology phenotype, with vacuoles unevenly distributed throughout the cells in contrast to the WT (Figure 4D). FB_1_ production assay showed that FB_1_ levels were repressed about 40 folds in ∆Fvvam7 and 20 folds in ∆Fvrab7 compared to the wild-type progenitor (Figure 4B). We tested the expression of 15 PKS genes through RT-qPCR to further verify the role of FvVam7 and FvRab7 in secondary metabolism (Table S2). As expected, almost all tested mRNA levels of PKS genes were altered in ∆Fvvam7 and ∆Fvrab7 mutants. More importantly, *PKS11* (*FUM1*), which is required for the first step of FB_1_ biosynthesis, was remarkably downregulated in the absence of FvVam7 and FvRab7. Based on these results, we concluded that Fum1 localized in the vacuole and that proper functions of FvVam7 and FvRab7 are indispensable for Fum1 protein expression and FB_1_ production. 

### 3.4. Proper Microtubule Assembly Regulates Vacuole Structure and FB_1_ Biosynthesis 

**Figure 5:**
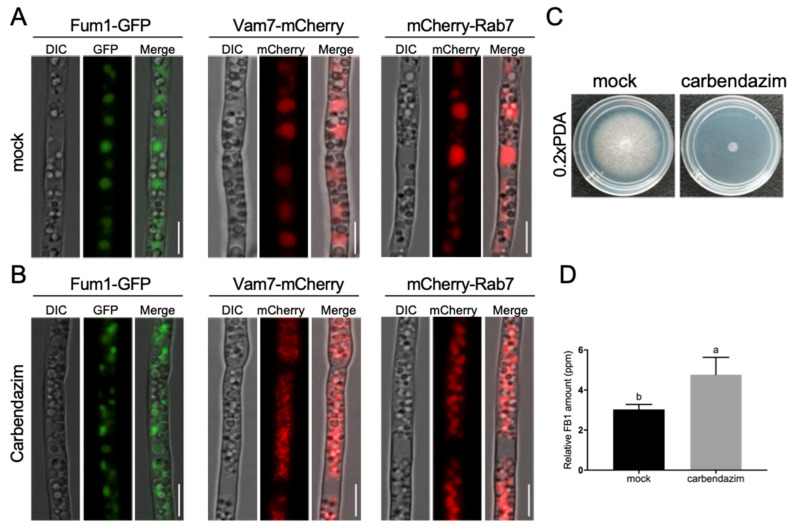
Carbendazim impacts on FB_1_ biosynthesis. Fum1-GFP, Vam7-mCherry, and Rab7-mCherry strains were inoculated in myro liquid medium without (**A**) and with (**B**) 1 μg/mL carbendazim treatment. Scale bar = 5 μm. (**C**) Sensitivity to carbendazim was shown by culturing WT on 0.2 × PDA or 0.2 × PDA + carbendazim (1 μg/mL) for 7 plates at 28 °C. (**D**) FB_1_ production in WT were tested after being cultured in myro broth with and without 1μg/mL carbendazim for 8 days of shaking at room temperature. Different letters on the bars indicated significance according to prism unpaired *t*-test at *p* < 0.05.

Previous research showed that MT cytoskeleton interacts with vacuole and regulates its morphology and function [30,31,32,33,34]. To investigate the roles of MT network in vacuole structure and Fum protein expression in *F. verticillioides*, carbendazim was used to treat Fum1-GFP, Vam7-mCherry, and mCherry-Rab7 strains to observe their localization and vacuole structure. Our findings showed that Fum1, Vam7, and Rab7 localized to the large vacuole structure in the mock treatment. However, with the treatment of 1.0 μg/mL carbendazim for 24 h, Fum1-GFP was localized to irregular and punctate structures in the cytoplasm, while Vam7-mCherry and mCherry-Rab7 exhibited small and fragmented vacuoles with irregular spheroids structures (Figure 5A,B). The results suggested that proper microtubule assembly is required for large vacuole structure and Fum1 localization. Furthermore, FB_1_ production level was significantly enhanced by 60% when treated with 1.0 μg/mL carbendazim compared to the mock-treated wild-type, while the mycelial growth was severely inhibited on 0.2 × PDA plate containing carbendazim (Figure 5C,D). Our results suggested that MT network plays important roles in regulating vacuole structure and FB_1_ biosynthesis in *F. verticillioides*.

### 3.5. Tubulin Fvα1 Is a Negative Regulator in FB_1_ Biosynthesis

**Figure 6:**
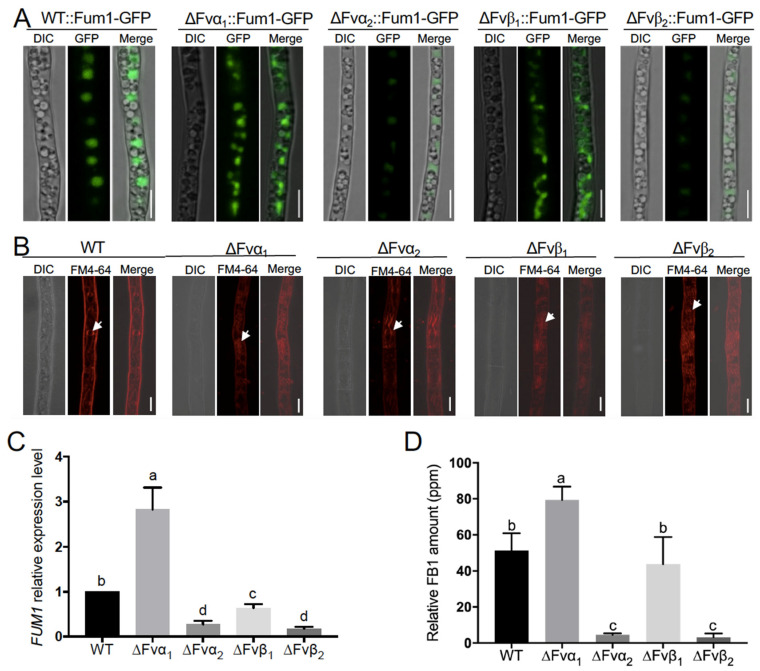
Fvα_1_ is negatively associated with *FvFUM1* expression and FB_1_ biosynthesis. (**A**) Fum1-GFP localization in WT, ΔFvα_1_, ΔFvα_2_, ΔFvβ_1_, and ΔFvβ_2_ strains. Scale bar = 5 μm. (**B**) FM4-64 was used to stain WT, ΔFvα_1_, ΔFvα_2_, ΔFvβ_1_, and ΔFvβ_2_ for vacuoles. Bar = 5 μm. (**C**) RT-qPCR results for *FvFUM1* under treatment of 1 μg/mL carbendazim relative to levels in myro broth. Significant differences were indicated with different letters on the bars according to prism two-way ANOVA at *p* < 0.05. (**D**) WT, ΔFvα_1_, ΔFvα_2_, ΔFvβ_1_, and ΔFvβ_2_ were cultivated on autoclaved corn kernels for 8 days at room temperature. FB_1_ levels were normalized with fungal ergosterol levels. Different letters on the bars indicated significance according to prism Ordinary one-way ANOVA multiple comparisons at *p* < 0.05.

**Figure 7:**
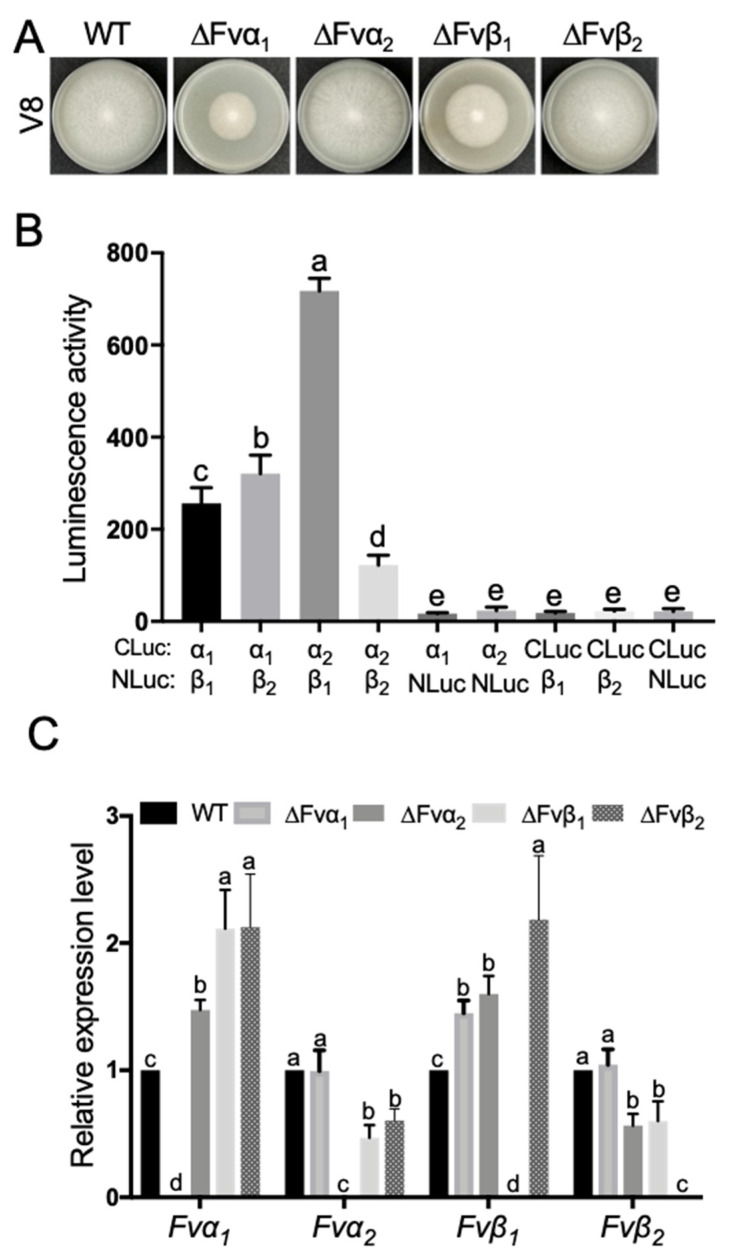
Growth and gene interaction assays using four microtubule deletion mutants. (**A**) WT, ΔFvα_1_, ΔFvα_2_, ΔFvβ_1_, and ΔFvβ_2_ strains were cultivated on V8 plates. (**B**) Luminescence activity was obtained from three replicates. Multiple negative controls (Fvα_1_-Cluc + NLuc, Fvα_2_-Cluc + NLuc, NLuc + Fvβ_1_-CLuc_,_ NLuc + Fvβ_2_-CLuc, NLuc-CLuc) were included in the study. Different letters on the bars indicated significance according to prism Ordinary one-way ANOVA multiple comparisons at *p* < 0.05. (**C**) *Fvα_1_*, *Fvα_2_*, *Fvβ_1_*, and *Fvβ_2_* relative expression in WT, ΔFvα_1_, ΔFvα_2_, ΔFvβ_1_, and ΔFvβ_2_ strains were determined by RT-qPCR. Significant differences were indicated with different letters on the bars according to prism two-way ANOVA at *p* < 0.05.

Carbendazim is a classical tubulin polymerization inhibitor. To better define the role of MT network in FB_1_ biosynthesis, four *F. verticillioides* tubulins, *Fvα_1_*, *Fvα_2_*, *Fvβ_1_*, and *Fvβ_2_*, were knocked out in Fum1-GFP background. Fum1-GFP signals in the four mutants and wild-type cultured in myro medium for 48 h were monitored. As shown in Figure 6A, Fum1-GFP were highly induced in ΔFvα_1_ and Δ*F*vβ_1_, while weak signals were observed in ΔFvα_2_ and ΔFvβ_2_ mutants. Examination of vacuole morphology in these tubulin mutants revealed that ΔFvα1 and ΔFvβ1 were unable to produce large vacuoles like the wild-type strain (Figure 6B). To verify these results, we tested the transcription levels of *FvFUM1* in wild-type and tubulin deletion mutants. The transcription level of *FvFUM1* was significantly increased in ΔFvα_1_ and dramatically reduced in ΔFvα_2_, ΔFvβ_1_, and ΔFvβ_2_, especially in ΔFvα_2_ and ΔFvβ_2_ (Figure 6C). Consistent with *FUM1* transcript level, FB_1_ level in ΔFvα_1_ was significantly higher, whereas that in ΔFvα_2_ and ΔFvβ_2_ were dramatically lower than the wild-type control (Figure 6D). Meanwhile, we observed a lower FB_1_ level in ΔFvβ_1_, although not statistically significant as *FUM1* transcript level. Further growth assay showed that the mutants ΔFvα_1_ and ΔFvβ_1_ exhibited significantly reduced growth rate (Figure 7A). To assess how these four proteins interact, we lastly performed split luciferase complementation assay in *F. verticillioides* under a FB_1_-producing condition to assess the interaction of these four proteins. Luciferase activity was detected in samples Fvα_1_:Fvβ_1_, Fvα_1_:Fvβ_2_, Fvα_2_:Fvβ_1_, and Fvα_2_:Fvβ_2_ (Figure 7B), which suggested that four types of tubulin heterodimer can be formed in *F. verticillioides* under FB_1_-producing condition. In addition, qRT-PCR revealed that the expression of other tubulin subunits was significantly upregulated in *Fvα_1_* and *Fvβ_1_* mutants (Figure 7C). Our results suggest that *Fvα_2_* and *Fvβ_2_* play a role in regulating FB1 biosynthesis through translocation of Fum enzymes to vacuole. However, more importantly, we concluded that Fvα_1_ serves as a negative regulator of FB_1_ production, but the mechanism associated with how Fvα_1_ deletion impacts Fum genes needs further examination.

## 4. Discussion 

*F. verticillioides* genetic and biochemical studies associated with fumonisin biosynthesis in the past three decades have provided valuable insights into how fumonisins are synthesized. However, the organization and compartmentation of fumonisin biosynthetic enzymes remained ambiguous. In this work, we tagged the first three Fum proteins in the proposed FB_1_ biosynthesis pathway. Under FB-producing conditions, we found that FvFum1, FvFum6, and FvFum8 localized to round, vacuole-like organelles. Further co-localization study demonstrated that FvFum1-GFP co-localizes with FvFum6-mCherry and FvFum8-mCherry in a live cell imaging assay, which suggested that these three Fum proteins occupy the same localization. Further characterization indicated that the organelle is the vacuole compartment since it showed positive localization with FM4-64. Genes involved in FB_1_ biosynthesis are highly arranged in gene clusters [16,17], and our results showed that proteins encoded by these genes are spatially well organized in vacuoles. Thus, we reasoned that *F. verticillioides* keeps those enzymes in the same organelle for optimum reaction and prevention of self-toxicity. However, the results of our localization study are different from a recent report which showed that Ds-Red::Fum8 localized in ER-derived vesicles under the control of constitutive *A. nidulans* promoter *PoliC* [35]. This report also showed that Fum17 and Fum18 share the same localization with Ds-Red::Fum8 with strong promoters *Pgln1* and *PoliC*, respectively. The different Fum proteins localizations in different organisms suggested that Fum proteins could be organism specific, or constitutive promoters may be a factor that leads to different localization. 

The vacuole is a dynamic cellular compartment that exerts many functions. Plant vacuoles were shown to permanently or temporarily store diverse and numerous secondary metabolites (>200,000 compounds) including chemicals used as defense or attractants [36]. In fungi, vacuoles are primarily recognized as a key site for degradation as well as for the storage of small molecules. To rule out misinterpretation of the Fum protein localization results, we carried out three additional experiments. We first conducted a time course study to monitor Fum protein localization. Fum proteins were localized in vacuole as well in earlier time points, but no fluorescence signal appeared until about 26 h incubation. Second, we used Syn1-GFP as a control which showed different localization pattern in the cell than Fum1-GFP [26]. Lastly, further experiment with glycine treatment, a major nitrogen, was also performed [37]. The results showed that Fum1-GFP localized to vacuoles, whereas fluorescence diminished after treatment of glycine. However, glycine treatment did not affect the localization of vacuole-related proteins Vam7-mCherry and mCherry-Rab7 (Figure S5). This is in line with our early finding that the addition of nitrogen repressed FB_1_ production [37]. Taken together, our results strongly support our novel finding that the key Fum proteins display vacuole localization in toxin-inducing broth. In addition, we expressed vacuole-associated proteins FvRab7 and FvVam7 with mCherry and analyzed their spatial relationship with FvFum1-GFP. In both *A. nidulans* and *F. gramminerum*, a small GTPase, Rab7, was shown to be localized to the vacuolar membrane [28,38]. We predicted that FvRab7 has the same localization since FvRab7 and FgRab7 are 100% identical in amino acid level. As expected, all these vacuole-associated proteins exhibited co-localization with FvFum1 under fumonisin-producing condition, demonstrating that vacuole is the organelle for fumonisin biosynthesis in *F. verticillioide*. However, we found that mCherry-FvRab7 co-localized with Fum1-GFP on the whole vacuole structure instead of appearing only on vacuolar membrane. To exclude the effect of incubation time on FvRab7 localization, we monitored the expression at 12h, 24h, 36h, and 48 h and found no mere vacuolar membrane localization (Figure S6). As a key FB_1_ biosynthesis level marker, Fum1-GFP expression was not detected in ΔFvrab7 mutant, and FB_1_ production was significantly reduced in the absence of Fvrab7. This result in *F. verticillioides* is in line with the data in *F. gramminerum* that ΔFgrab7 mutant showed significantly lower DON production [39]. However, in *A. parasiticus*, deletion mutant of *vb1* (Rab7 homolog) led to a 7-fold higher of aflatoxin compared to WT in an aflatoxin-inducing condition [7]. Those strongly revealed the functional importance of the vacuole in mycotoxin production.

We also examined other factors that may potentially influence vacuole’s function. As an important cytoskeleton, MT and tubulin heterodimers interact with a variety of cellular components (metabolic enzymes or cellular organelles), thereby regulating intracellular metabolic pathways. Evidence revealed that MT binds to most of the glycolytic enzymes and regulates their activities [40,41,42]. In HeLa cell, MT is crucial for the spatial distribution and biological functions of purinosomes [43]. Our recent study demonstrated that MT participated in toxisomes formation and DON biosynthesis in *F. graminearum* [44]. In this work, we analyzed the regulatory role of MT network in vacuole morphology via carbendazim treatment. The results demonstrated that fragmented vacuoles were observed in hyphae under carbendazim treatment with 1.2 μg/mL (100% inhibition against mycelial growth of *F. verticillioides*) for 24 h in *F. graminearum*. This is consistent with vacuole localization in *F. verticillioides.* We found that MT network participated in vacuole morphology regulation in *F. verticillioides*. Clear evidence was also found in some plant species that vacuole morphology is regulated by MT [30,34].

Interestingly, we found that FB_1_ production significantly increased when treated with carbendazim in *F. verticillioides*. However, the expression of *FvFum1* showed no significant difference when compared to the control group by RT-qPCR analysis. The reason is still unclear. In *A. parasiticus*, two important aflatoxin synthetic enzymes were localized in vesicles. When deleting *vb1* (a member of Vps tethering complex) in *A. parasiticus* or using Vps tethering complex inhibitor Sortin3 to treat *A. parasiticus*, fragmented vacuoles and increased aflatoxin production were observed, but the expression of aflatoxin biosynthetic genes showed no significant changes in *vb1* mutants or under Sortin3 treatment [7]. We speculated that fragmented vacuoles could facilitate FB_1_ biosynthesis, yet there is no close correlation between the expression levels of *FvFum1* and vacuole morphology in *F. verticillioides*. Additionally, in *F. graminearum*, low concentrations of carbendazim boosted DON production, but high concentrations significantly reduced it [44]. Our experiments utilized a 1 μg/mL carbendazim concentration, resulting in increased FB1 production. The effects of carbendazim on FB1 production in *F. verticillioides* could also relate to the concentrations of carbendazim.

Our previous study showed that α1-β2 tubulin heterodimer is critical for toxisome formation and DON biosynthesis in *F. graminearum* [44]. However, gene deletion assays in *F. verticillioides* revealed that three tubulin subunits were involved in FB_1_ biosynthesis. ΔFvα1 showed significantly increased FB_1_ production, whereas FB_1_ production in ΔFvα2 and ΔFvβ2 mutants was dramatically reduced. The differences between *F. verticillioides* and *F. graminearum* indicated that the regulatory roles of tubulin subunits in secondary metabolism vary among species. Further research is needed to unveil the different FB_1_ regulating mechanisms in *F. verticillioides.*

In summary, this report revealed the localization of Fum proteins in vacuole. Two vacuole proteins, FvRab7 and FVam7, are required for FB1 biosynthesis. Further study showed that disrupted MT assembly caused the change in Fum1 localization and enhanced FB_1_ production. Fvα1 tubulin is a negative regulator in FB_1_ production. However, further studies are needed to better understand the effect of MT on promoting FB_1_ production and how the protein–protein interaction network of tubulins interact with MT in regulating FB_1_ production. 

## Data Availability

All data generated or analyzed in this study are included in this published article and its supplementary information.

## Supplementary Materials

The following supporting information can be downloaded at: https://www.mdpi.com/article/10.3390/jof9020268/s1, Figure S1: Schematic description and confirmation of the split marker strategy to generate Fum1 GFP strain. Figure S2: Schematic representation of the gene deletion and mutant screening. Figure S3: Localization of Syn1-GFP in myro and MM broth. Figure S4: Fum1-GFP and Fum8-GFP absent localization on growing apical area. Figure S5: Nitrogen repression of Fum1-GFP expression. Figure S6: Time courses of Vam7-mCherry and mCherry-Rab7. Table S1: Primers used in this study. Table S2: Relative expression level of PKS genes in wild-type, ΔFvrab7 and ΔFvvam7.
