# Supplementary material for: Vacuole Proteins with Optimized Microtubule Assembly Is Required for Fum1 Protein Localization and Fumonisin Biosynthesis in Mycotoxigenic Fungus Fusarium verticillioides
Source: J Fungi (Basel). 2023 Feb 16;9(2):268. doi: 10.3390/jof9020268 (PMC9961181; doi:10.3390/jof9020268)
Supplement: Supplementary file 1 [file jof-09-00268-s001.zip › jof-2127902-supplementary.pdf]

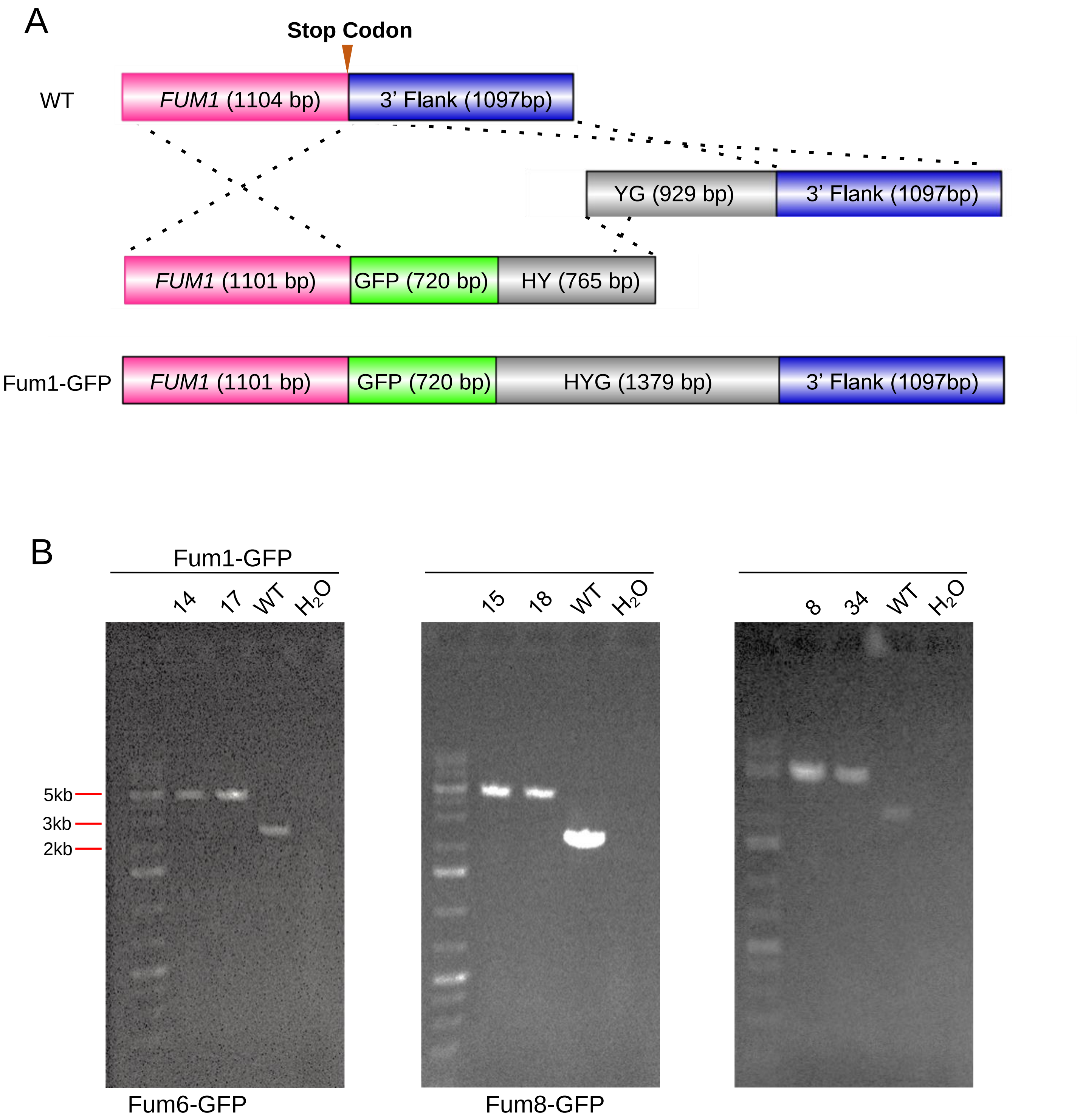

Figure S1. Schematic description and confirmation of the split marker strategy to generate Fum1 GFP strain. (A) Schematic of the GFP and split hygromycin B phosphotransferase gene (*HPH*) were to replace the *FvFUM1* gene stop codon. (B) PCR confirms the generation of two independent single insertion fluorescent strains. The PCR bands in the correct fluorescent strains are around 4.5kb compared to about 2.5kb in wild type strains.

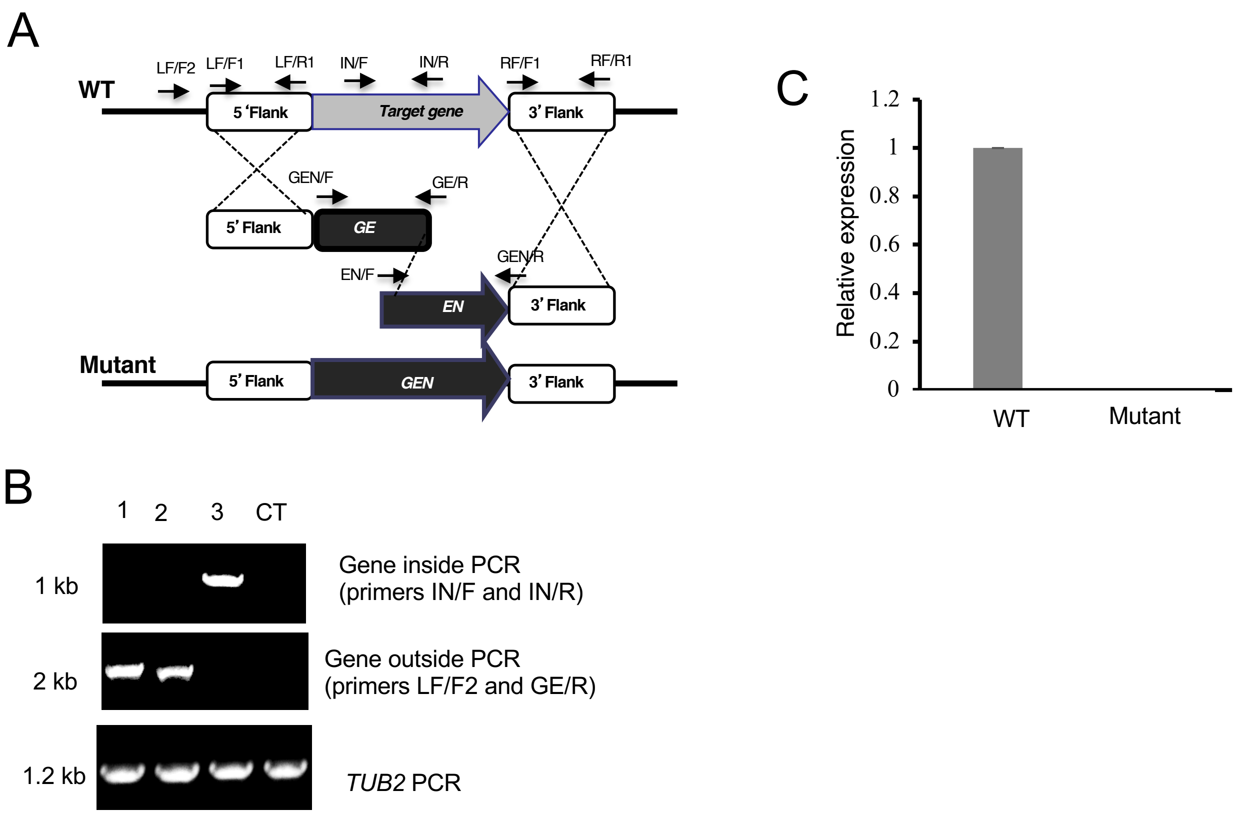

Figure S2. Schematic representation of the gene deletion and mutant screening. (A) Target replacement of targeted gene with the geneticin gene *(GEN)* by the split marker technique through homologous recombination. Arrows indicate primers used for PCR. *GE, GEN* 5' partial amplicon, *EN*, *GEN* 3' partial amplification. (B) PCR conformation of gene knockout mutants. Strains 1 and 2 are example of knockout mutants, and strain 3 is an example of a false positive. CT is negative control PCR with no DNA template. Beta-tubulin gene (*TUB2*) was PCR amplified as positive control. (C) One of the knockout mutants was further confirmed by qPCR. All primers used are listed in Table S1.

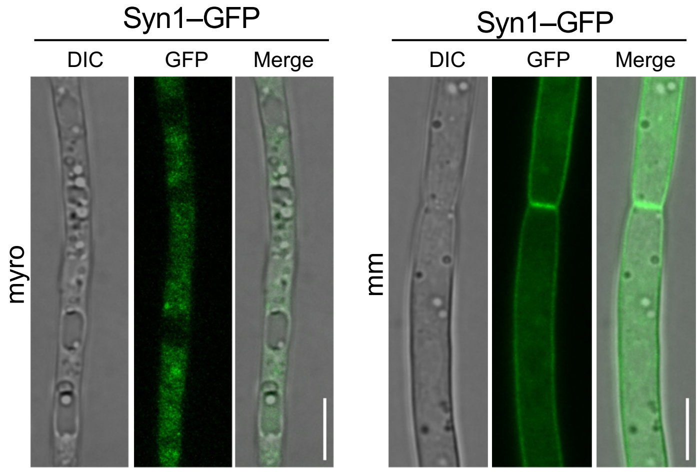

Figure S3. Localization of Syn1-GFP in myro and MM broth. Syn1-GFP subcellular images in myro and MM medium were used a control. Bar = 5 μm.

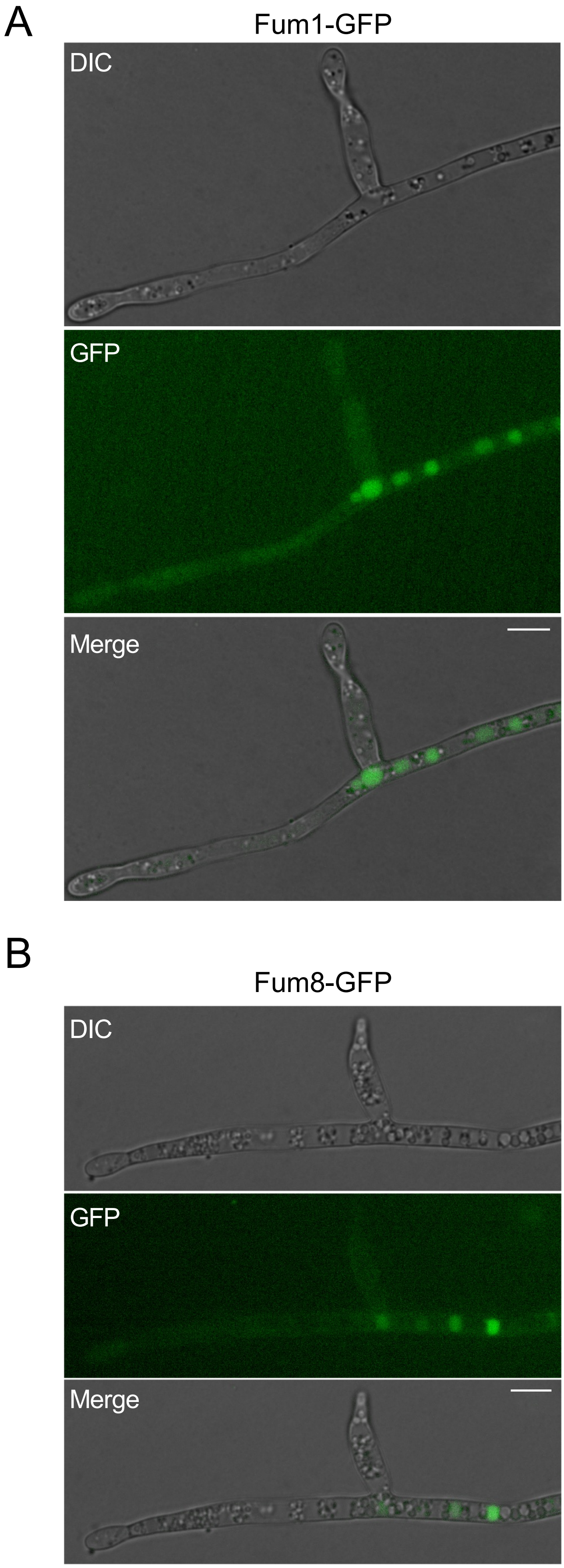

Figure S4. Fum1-GFP and Fum8-GFP absent localization on growing apical area. The Fum1-GFP (A) and Fum8-GFP (B) signals cannot be detected in apical and branching hyphae. Bar = 5 μm.

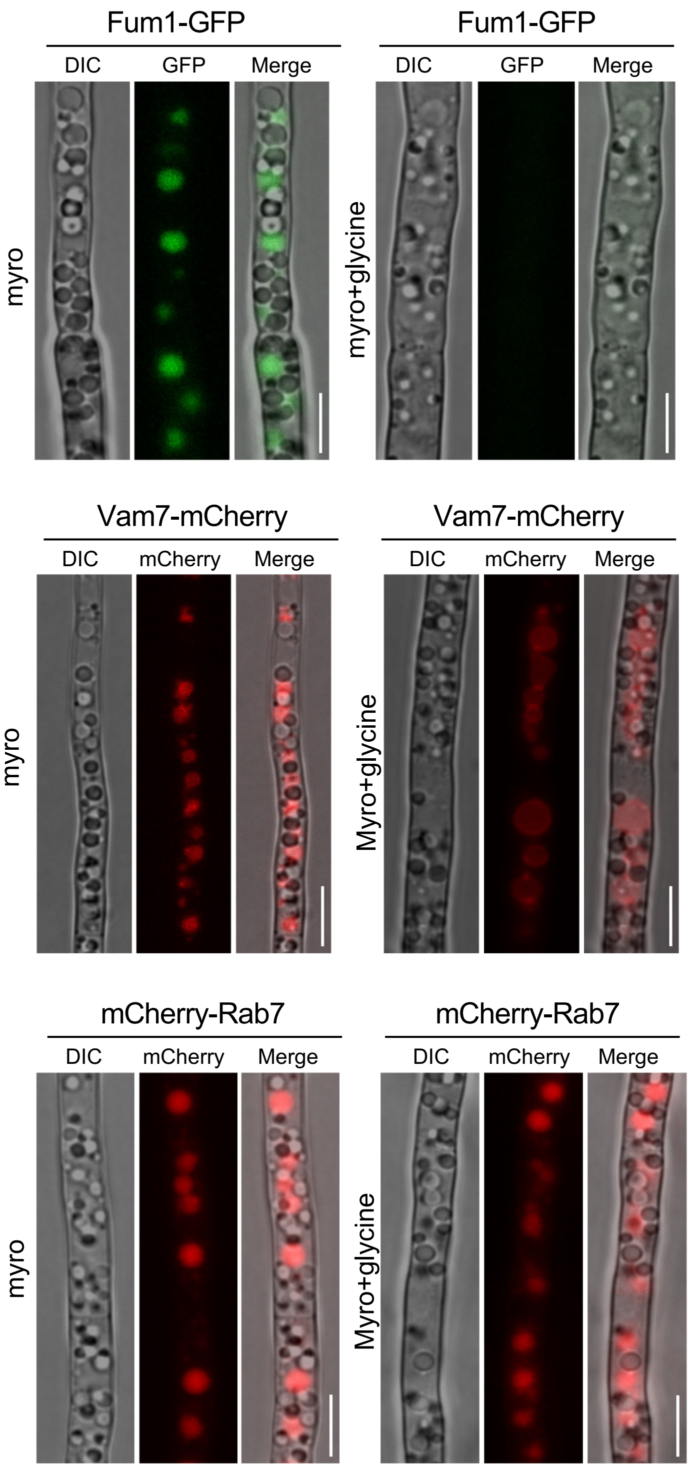

Figure S5. Nitrogen repression of Fum1-GFP expression. Fum1-GFP strain conidia were grown in myro liquid medium with constant shaking. After 48 h incubation, the culture was separated into two flasks without and with adding glycine to final concentration 10 μM in myro liquid medium. Images were taken after 5 more hours of incubation. Vam7-mCherry and mCherry-Rab7 were as controls. Bar = 5 μm.

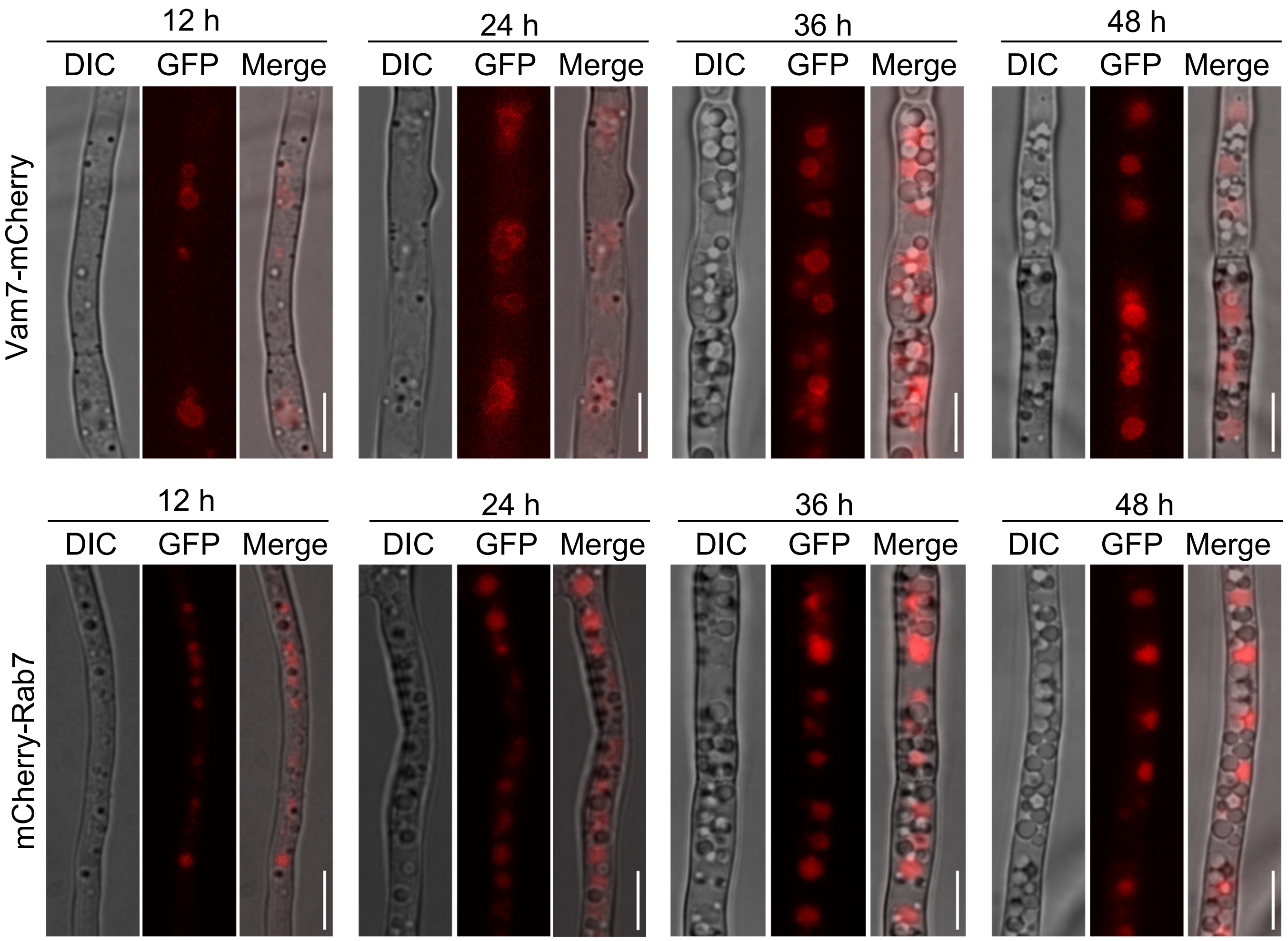

Figure S6. Time courses of Vam7-mCherry and mCherry-Rab7. Examination of Vam7-mCherry and mCherry-Rab7 at four different time points (12 h, 24 h, 36 h, 48 h) in myro liquid medium. Bar = 5 μm.

Table S1. Primers used in this study.

Primer	Primer sequence (5’-3’)	Application
FUM1-LF2	TCC TAT CAC ACC AAA GCC TCT AC	validation of Fum1-GFP strain
FUM1-LF1	GGT GCC CGA TAC CTA ATC TTC TTC	amplify *FUM1* 5’ flank sequence
FUM1-LR1	GCT CCT CGC CCT TGC TCA CCA TTG GGG CCT TCA TTT CTA GAT ACG	amplify *FUM1* 5’ flank sequence (Fum1-GFP)
FUM1-RF1	ATA GAG TAG ATG CCG ACC GGG AAC ATA CCT ACC TAG ACA CTG AGC TGG	amplify *FvFUM1* 3’ flank sequence (*HPH* linker)
FUM1-RR1	CTC CGG CCA CTC AAG GAA TAA A	amplify *FvFUM1* 3’ flank sequence
FUM1-RR2	GTC TCA CTG AGC CTG AGA GAT T	validation of Fum1-GFP strain
FUM6-LF2	GCG ACC ACG TCT ATA TCC TGC	validation of Fum6-GFP strain
FUM6-LF1	TTC CGT TAG ATA CGC CCA TCA C	amplify *FUM6* 5’ flank sequence
FUM6-LR1	GCT CCT CGC CCT TGC TCA CCA TCA CAT AAA CTT CTT CCA CGT ATC TCG	amplify *FUM6* 5’ flank sequence (Fum6-GFP)
FUM6-RF1	ATA GAG TAG ATG CCG ACC GGG AAC GTT GGG CTC TTC GGG TGC ATT	amplify *FvFUM6* 3’ flank sequence (*HPH* linker)
FUM6-RR1	ATT GGT CTA CGG AGC GAC AAA G	amplify *FvFUM6* 3’ flank sequence
FUM6-RR2	TCT AGC CAA GAA CCC AGA ACG	validation of Fum6-GFP strain
FUM8-LF2	CGC TAC GCC ATT ATG GTA AGA AC	validation of Fum8-GFP strain
FUM8-LF1	GCA TCA CCG CCA CTG TCT TTA C	amplify *FUM8* 5’ flank sequence
FUM8-LR1	GCT CCT CGC CCT TGC TCA CCA TAC ATG TCC CTC GCG ATA AAA TCT	amplify *FUM8* 5’ flank sequence (*HPH* linker)
FUM8-RF1	ATA GAG TAG ATG CCG ACC GGG AAC ATG TAA CTA GGC AGA GTA GTA GTA TTG G	amplify *FvFUM8* 3’ flank sequence (Fum8-GFP)
FUM8-RR1	TAA ATG TGC CCA CTA CAC TAT CCG	amplify *FvFUM8* 3’ flank sequence

FUM8-RR2	TGC TGG CTG CAA ACA ATG TCA	validation of Fum8-GFP strain
GFP-F	ATG GTG AGC AAG GGC GA	amplify GFP fragment
GFP-R	TTG ACC TCC ACT AGC TCC AGC CAA GTT ACT TGT ACA GCT CGT CCA TGC C	amplify GFP fragment
HYG/F	TTG GCT GGA GCT AGT GGA GGT CAA	amplify HY fragment
HY/R	GTA TTG ACC GAT TCC TTG CGG TCC GAA	amplify HY fragment
HYG/R	GTT CCC GGT CGG CAT CTA CTC TAT	amplify YG fragment
YG/F	GAT GTA GGA GGG CGT GGA TAT GTC CT	amplify YG fragment
FUM6_LR (mcherry)	CT CCT CGC CCT TGC TCA CCA T CAC ATA AAC TTC TTC CAC GTA TCT CG	amplify *FUM6* 5' flank sequence (Fum6-mCherry)
FUM6_RF(GEN)	ATTCCACACAACATACGAGCC GTT GGG CTC TTC GGG TGC ATT	amplify *FUM6* 5' flank sequence (Fum6-mCherry)
FUM8_LR (mcherry)	CT CCT CGC CCT TGC TCA CCA TAC ATG TCC CTC GCG ATA AAA TCT	amplify *FUM8* 5' flank sequence (Fum8-mCherry)
FUM8_RF(GEN)	ATTCCACACAACATACGAGCCATG TAA CTA GGC AGA GTA GTA GTA TTG G	amplify *FUM8* 5' flank sequence (Fum8-mCherry)
mCherry-F	ATGGTGAGCAAGGGCGAGGAG	amplify mCherry fragment
mCherry-R	TGG CGT TAC CCA ACT TAA TCG CTA CTT GTA CAG CTC GTC CAT GCC	amplify mCherry fragment
Vam7_mcherry-F	agggaacaaaagctgggtacc AGC TGA CAG CCT GTG GAA TAA	construction of pKNT-Vam7-mCherry
Vam7_mcherry-R	gcccttgctcaccataagctt CAT TTT CTT GAT CCG GTT GTT CGC	construction of pKNT-Vam7-mCherry
Rab5-Pro-F	agggaacaaaagctgggtacc TCATTGTAGTTGGCGACGTGTC	amplify *FvRAB5* promoter sequence
Rab5-Pro-R	CTC CTC GCC CTT GCT CAC CAT CGT AGT GAG AGA TGG GAA TGT AAT TTC G	amplify *FvRAB5* promoter sequence
Rab5-ORF-F	GGC ATG GAC GAG CTG TAC AAG ATG GCC TCT CGA CAA CCT CCA	amplify *FvRAB5* sequence
Rab5-ter-R	tcagtaacgttaagtggatcc AGA CCT CGG ACC AGA GCC TAA A	amplify *FvRAB5* sequence
Rab7-Pro-F1	agggaacaaaagctgggtacc AAT ACA GTC AAC GGC TGG AC	amplify *FvRAB7* promoter sequence
Rab7-Pro-R1	CTC CTC GCC CTT GCT CAC CAT CGT GTT GAA ATA TTG TTT CTG GAC GAG	amplify *FvRAB7* promoter sequence
Rab7-ORF-F1	GGC ATG GAC GAG CTG TAC AAG ATG TCT TCA CGA AAG AAG GTC CTT	amplify *FvRAB7* sequence

Rab7-Ter-R1	tcagtaacgttaagtggatcc GGT GGA TGT AAT AAG GGA GTG TAG	amplify *FvRAB7* sequence
Rab11-Pro-F	agggaacaaaagctgggtacc AGG TGT AGG TGT AGG TAG GTA GGT	amplify *FvRAB11* promoter sequence
Rab11-Pro-R	CTC CTC GCC CTT GCT CAC CAT CGT GGC TAC GGT GGT GTT GCT TCA	amplify *FvRAB11* promoter sequence
Rab11-ORF-F	GGC ATG GAC GAG CTG TAC AAG ATG GCC AAC GAC GAA TAT GAT	amplify *FvRAB11* sequence
Rab11-ter-R	tcagtaacgttaagtggatcc GAT AGT AGA GCA ACA AGA CCA CC	amplify *FvRAB11* sequence
Vam7_LF/F2	AGC TGA CAG CCT GTG GAA TAA	validation of *FvVAM7* deletion
Vam7_LF/F1	CGG TTT GGG TGT TGT GTA TGA T	amplify *FvVAM7* 5' flank sequence
Vam7_LF/R1	G GCG TTA CCC AAC TTA ATC G GTT GAT TGA AGC TTG GTT GTG GT	amplify *FvVAM7* 5' flank sequence
Vam7_RF/F1	T TCC ACA CAA CAT ACG AGC C CAG CTA CCT CTC CTA CTA CTG GTA	amplify *FvVAM7* 3' flank sequence
Vam7_RF/R1	CAT TAT TCA CTG CGT GGC TCC	amplify *FvVAM7* 3' flank sequence
Vam7_IN/F	TCA CTC TTC GAC TAC CTC TGC	validation of *FvVAM7* deletion
Vam7_IN/R	TCG TCG AAT AAC TGC AGT GAG C	validation of *FvVAM7* deletion
Vam7_RF/R2	AGG AGT GGC TGT CCG TTC ATC	validation of *FvVAM7* deletion
Rab7_LF/F2	GTA GAG GCA GTA TCG GCA TCA TT	validation of *FvRAB7* deletion
Rab7_LF/F1	AGA TAC GAC GGG TAC GTA GAA GT	amplify *FvRAB7* 5' flank sequence
Rab7_LF/R1	G GCG TTA CCC AAC TTA ATC G GTT GAA ATA TTG TTT CTG GAC GAG AAG C	amplify *FvRAB7* 5' flank sequence
Rab7_RF/F1	T TCC ACA CAA CAT ACG AGC C AGG ATG ACA TGG GCA GAT GCT	amplify *FvRAB7* 3' flank sequence
Rab7_IN/F	AAG AAG GTC CTT CTC AAG GTG CG	validation of *FvRAB7* deletion
Rab7_IN/R	GCT TTG TCA AGG TGT CAG AGC	validation of *FvRAB7* deletion
Rab7_RF/R1	GAT ACA GTT GCG CTG TGT GAA AG	amplify *FvRAB7* 3' flank sequence
Rab7_RF/R2	CCG TAC TCG AGG TTG GTG TAG	validation of *FvRAB7* deletion

GEN/F	CGA TTA AGT TGG GTA ACG CCA G	amplify GE fragment
GE/R	ATC ACG GGT AGC CAA CGC TA	amplify GE fragment
EN/F	TCG ACC ACC AAG CGA AAC AT	amplify EN fragment
GEN/R	GGC TCG TAT GTT GTG TGG AAT T	amplify EN fragment
Vam7-qpcr-F	ATT GGG CAA CGA GGG AGT GCT A	qPCR analysis
Vam7-qpcr-R	CCT CTT CAT GGA TTC GCT CTC CCA	qPCR analysis
Rab7-qpcr-F	GGA CCC TCC CAA CTT CCC ATT T	qPCR analysis
Rab7-qpcr-R	TTG GAC TGG CAG AAT GTC ATG GC	qPCR analysis
α1-qpcr-F	ACTACGGCAAGAAGAGCAAG	qPCR analysis
α1-qpcr-R	GTGGTGTGTGTGGTAAGGATAG	qPCR analysis
α2-qpcr-F	CTTCAGGGTTTCCTGATCTTCC	qPCR analysis
α2-qpcr-R	GCTTAGACTTCTTGCCGTACTC	qPCR analysis
β1-qpcr-F	TATGAAGGAGGTCGAGGATCAG	qPCR analysis
β1-qpcr-R	CAAAGGGCTGTCTGGATGTT	qPCR analysis
β2-qpcr-F	GCAGGGCTTCCAACATCTTA	qPCR analysis
β2-qpcr-R	TATCGACCGTTGCGGAAATC	qPCR analysis
β1 nLuc F	AAGCTCGAGTAGTCGACATGCGTGAGATTGTAAGTACC	construction of pFNLuc-*Fvβ*$_1$
β1 nLuc R	CGTACGAGATCTGGTCGACCTCCTCGCCCTCAGGGAGCT	construction of pFNLuc-*Fvβ*$_1$
β2 nLuc F	AAGCTCGAGTAGTCGACATGCGTGAGATTGTGAGACT	construction of pFNLuc-*Fvβ*$_2$
β2 nLuc R	CGTACGAGATCTGGTCGACGCCCTCATACTCCTCAGGCT	construction of pFNLuc-*Fvβ*$_2$
α1 cLuc F	CGTCCCGGGGCGGTACCCGTGAGGTCATTAGCATCAA	construction of pFCLuc-*Fvα*$_1$
α1 cLuc R	TTGGATCCCCGGGTACCTTAGTACTCAGCCTCGAGCT	construction of pFCLuc-*Fvα*$_1$
α2 cLuc F	CGTCCCGGGGCGGTACCAAGGGCGAGGTATGTCTAC	construction of pFCLuc-*Fvα*$_2$

α2 cLuc R	TTGGATCCCCGGGTACCCTAGTACTCGAGCTCCTCTT	construction of pFCLuc-$Fv\alpha_2$
α1 LF/F1	CTTACGAACGCAATGGAAT	amplify $Fv\alpha_1$ 5’ flank sequence
α1 LF/R1	GGCGTTACCCAACTTAATCGGATTGGACCTTCTATGGATT	amplify $Fv\alpha_1$ 5’ flank sequence
α1 RF/F1	TTCCACACAACATACGAGCCCTAATGAAGTCCGCGAATGT	amplify $Fv\alpha_1$ 3’ flank sequence
α1 RF/R1	GCCGTATGTATCGTGGAAG	amplify $Fv\alpha_1$ 3’ flank sequence
α1 IN/F	TCAAGGTTTCCTCGTGTTC	validation of $Fv\alpha_1$ deletion
α1 IN/R	TCGCCGTTAGGCACATTCT	validation of $Fv\alpha_1$ deletion
α1 LF/F2	TGCTATGGCACAATCCGTC	validation of $Fv\alpha_1$ deletion
α1 RF/R2	GAACTGCTTTGCCTACGGT	validation of $Fv\alpha_1$ deletion
α2 LF/F1	CTGGAGTCAAGGAGGATGG	amplify $Fv\alpha_2$ 5’ flank sequence
α2 LF/R1	GGCGTTACCCAACTTAATCGGAGGTGGTGATGGTTTTCG	amplify $Fv\alpha_2$ 5’ flank sequence
α2 RF/F1	TTCCACACAACATACGAGCCATAGGCATTGAATACATTAGCC	amplify $Fv\alpha_2$ 3’ flank sequence
α2 RF/R1	GCCGCAGAACTCAATGGTA	amplify $Fv\alpha_2$ 3’ flank sequence
α2 IN/F	CTCAACGCTAACCGCACAA	validation of $Fv\alpha_2$ deletion
α2 IN/R	AAGCCTATGGGGAGTCAGT	validation of $Fv\alpha_2$ deletion
α2 LF/F2	CGCCATCACAAGAACAAAG	validation of $Fv\alpha_2$ deletion
α2 RF/R2	CGCCAAAGACAACAAATCG	validation of $Fv\alpha_2$ deletion
β1 LF/F1	AGGGGTTGTGCCTCTTTTC	amplify $Fv\beta_1$ 5’ flank sequence
β1 LF/R1	GGCGTTACCCAACTTAATCGGAGACCAAAGGGCGATGTT	amplify $Fv\beta_1$ 5’ flank sequence
β1 RF/F1	TTCCACACAACATACGAGCCCTGACTTACTCGAACTGGTC	amplify $Fv\beta_1$ 3’ flank sequence
β1 RF/R1	AGGGAAAATGAGTAGGAAGAC	amplify $Fv\beta_1$ 3’ flank sequence
β1 IN/F	GTCACCACCTGTCTCCGTT	validation of $Fv\beta_1$ deletion
β1 IN/R	CTCCATCTCGTCCATACCC	validation of $Fv\beta_1$ deletion
β1 LF/F2	GGTGGTTTATGGGTGTTGA	validation of $Fv\beta_1$ deletion
β1 RF/R2	ATTCACGGAAAGCGTCTCG	validation of $Fv\beta_1$ deletion

β2 LF/F1	ACCTCGCTCTGAGAATACC	amplify *Fv*β_2 5’ flank sequence
β2 LF/R1	GGCGTTACCCAACTTAATCGGGCTTGATTGAGGCTTTGC	amplify *Fv*β_2 5’ flank sequence
β2 RF/F1	TTCCACACAACATACGAGCCTCTCTTTAATCATGCTTGACG	amplify *Fv*β_2 3’ flank sequence
β2 RF/R1	GAAACTACGACCGCAGCAT	amplify *Fv*β_2 3’ flank sequence
β2 IN/F	CATCTTCAGGGTTTCCAGC	validation of *Fv*β_2 deletion
β2 IN/R	CTTGAGCATCTGGTCTTCG	validation of *Fv*β_2 deletion
β2 LF/F2	TCACGGTGCCTGAAAAGTC	validation of *Fv*β_2 deletion
β2 RF/R2	ACCTCCGTATCAGCCAAAC	validation of *Fv*β_2 deletion

The sequence complementary to a DNA fragment or a linearized vector was underlined. The restriction site was gray shading.

Table S2. Relative expression level of *PKS* genes in wild-type, ΔFvrab7 and ΔFvvam7.

	Relative expression level		
Gene	WT	ΔFvrab7	ΔFvvam7
PKS1	1	5.64±0.0.77	3.45±0.63
PKS2	1	0.32±0.07	0.25±0.11
PKS3	1	1.36±0.17	420.7±32.8
PKS4	1	0.13±0.002	0.57±0.07
PKS5	1	0.30±0.11	0.39±0.19
PKS6	1	1.91±0.23	1.82±0.17
PKS7	1	0.08±0.03	0.25±0.08
PKS8	1	0.17±0.09	0.65±0.19
PKS9	1	3.32±0.49	1.91±0.28
PKS10	1	1.90±0.33	0.93±0.27
PKS11	1	0.12±0.03	0.17±0.04
PKS12	ND	ND	ND
PKS13	1	0.44±0.24	0.46±0.05
PKS14	1	1.40±0.10	1.32±0.14
PKS15	1	2.10±0.37	2.32±0.41

Mycelia of each mutants were incubated in myro liquid medium for 5 days at 28℃. Each gene expression was normalized with β-tubulin expression level. Gene expressions were calculated using $2^{-\Delta\Delta Ct}$. Gene expression level of wild-type strain was standardized to 1.0. Three replicates were performed for each test. ND: not detected.
